# Sagittal sinus thrombosis due to L-asparaginase

**DOI:** 10.4103/1817-1745.66683

**Published:** 2010

**Authors:** Nisar A. Wani, Tasleem Kosar, Nazir A. Pala, Umar A. Qureshi

**Affiliations:** Department of Radiodiagnosis and Imaging, Sher-I-Kashmir Institute of Medical Sciences (SKIMS), Srinagar, J & K, India; 1 Department of General Medicine, Sher-I-Kashmir Institute of Medical Sciences (SKIMS), Srinagar, J & K, India; 2 Department of Pediatrics and Neonatology, Sher-I-Kashmir Institute of Medical Sciences (SKIMS), Srinagar, J & K, India

**Keywords:** Cerebral sinovenous thrombosis, L-asparaginase, leukemia, venous infarct

## Abstract

Cerebral Sinovenous Thrombosis (CSVT) is a serious complication of L-asparaginase chemotherapy for leukemia in children. Clinical features of headache, altered consciousness, focal neurological deficit, and seizures developing during or immediately after treatment with L-asparaginase should alert the treating physician to the possibility of CSVT. Immediate imaging of the brain should be done using CT and MRI and the veins should be visualized noninvasively by CT and MR venography. We report two children on induction therapy for acute leukemia who presented with seizures, headache, and altered consciousness. Venous infarcts with and without hemorrhage were seen on CT in one patient and the empty delta sign was seen after contrast injection; however, the early changes were missed by CT. MRI detected dural sinus thrombosis relatively earlier in another patient, while the CT findings were equivocal; in this patient, contrast-enhanced MRI showed the empty delta sign and MR venography confirmed absent flow in the superior sagittal sinus, which was diagnostic of sinus thrombosis. Rapid anticoagulation was started with heparin and maintained with warfarin. The child with a unilateral small nonhemorrhagic infarct made a complete recovery while the other, with bilateral hemorrhagic infarcts, did not survive. We stress the importance of early diagnosis of CSVT using CT and MRI in children with leukemia being treated with L-asparaginase; this will permit timely treatment.

## Introduction

Cerebral sinovenous thrombosis (CSVT) is a rare disorder in children. Its incidence is declining as some of the conditions historically associated with CSVT (such as mastoiditis) are now treatable.[1] However, CSVT is being increasingly diagnosed now because of greater awareness among clinicians, availability of advanced neuroimaging techniques, and the survivalof children with previously lethal diseases that confer a predispositionto sinovenous thrombosis.[1] One such predisposing condition is intensive induction treatment of leukemia. The treatment of patients with childhood Acute Lymphoblastic Leukemia (ALL) has extended the 5-year event-free survival rates and, consequently, the morbidities secondary to treatment for ALL have assumed increasing importance.[2] Thromboembolic events, including CSVT, are among the more frequent and serious complications of ALL and its treatment.[2,3] The timing of thrombosis in children with ALL is very consistent in the literature, occurring either during or immediately after chemotherapy with L-asparaginase.[2,3]

## Case Reports

### Case 1

**Figure 1:**
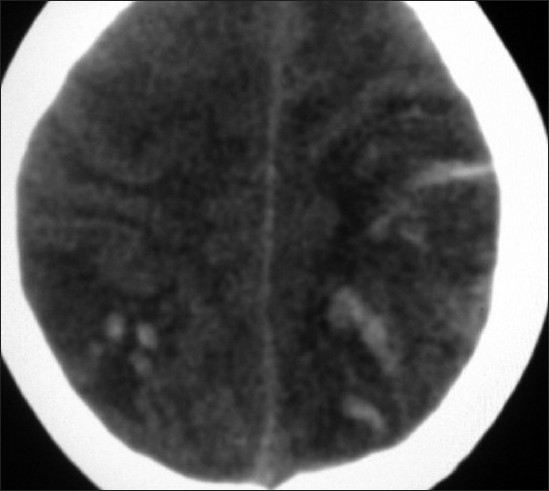
Noncontrast transverse CT image of the brain showing venous infarcts with hemorrhage in the bilateral parietal regions of the cerebral hemispheres

**Figure 2:**
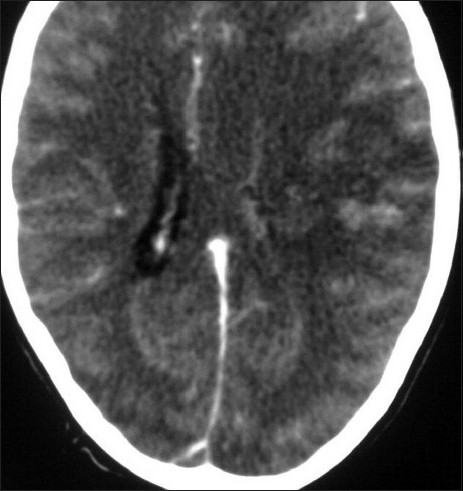
Contrast-enhanced transverse CT image showing the empty delta sign in the superior sagittal sinus posteriorly, with a large infarct in the lefthemisphere causing a midline shift to the right

A 10-year-old boy with newly diagnosed ALL, receiving intravenous vincristine and doxorubicin as well as oral prednisolone and intrathecal methotrexate, was now on treatment with intravenous l-asparaginase. He reported headache while on treatment with L-asparaginase. On examination, Kernig’s sign was absent and there was no papilledema. The initial plain CT did not reveal any definite focal lesion in the brain. Two days later, his headache worsened and he developed vomiting followed by seizures and loss of conciousness. He was resuscitated and anticonvulsant medication was given intravenously. Noncontrast CT now showed hemorrhagic infarcts in the bilateral high parietal regions [Figure 1]; the infarcts were larger on the left side, with a mass effect and midline shift toward the right [Figure 2]. Contrast-enhanced CT showed hypodense attenuation of the superior sagittal sinus posteriorly with peripheral enhancement (called the empty delta sign), Which was suggestive of sinus thrombosis [Figure 2]. The patient’s coagulogram was significantly altered, with antithrombin level of 50% and fibrinogen level of 0.9 g/l; the Prothrombin Time (PT) and Activated Partial Thromboplastin Time (APTT) were abnormal. L-asparaginase was stopped and fresh frozen plasma was infused in an attempt to reverse L-asparaginase-induced antithrombin deficiency. Phenytoin injections were continued for control of seizures. However, the patient continued to deteriorate and the coagulogram abnormalities could not be reversed; he expired 2 days after diagnosis of superior sagittal sinus thrombosis complicated by venous infarction.

### Case 2

**Figure 3:**
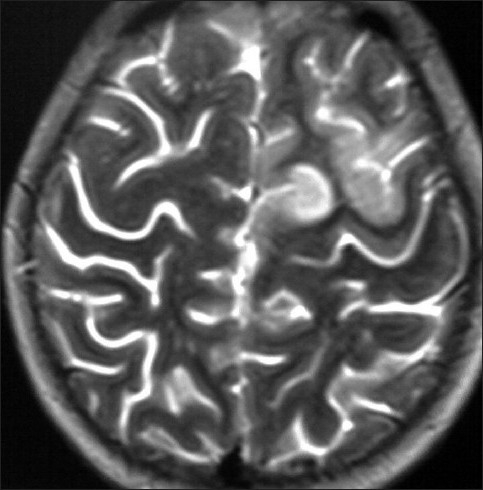
T2-weighted axial MR image showing a small area of subcortical white matter edema in the left high parietal parasagittal region due to a venous infarct

**Figure 4:**
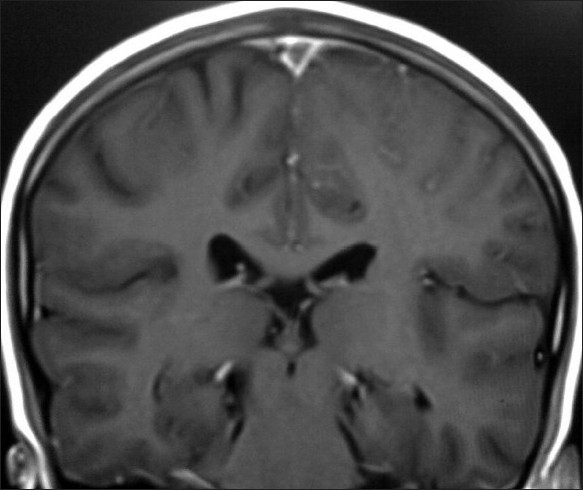
Coronal T1-weighted postcontrast MR image showing empty delta sign due to superior sagittal sinus thrombosis

**Figure 5:**
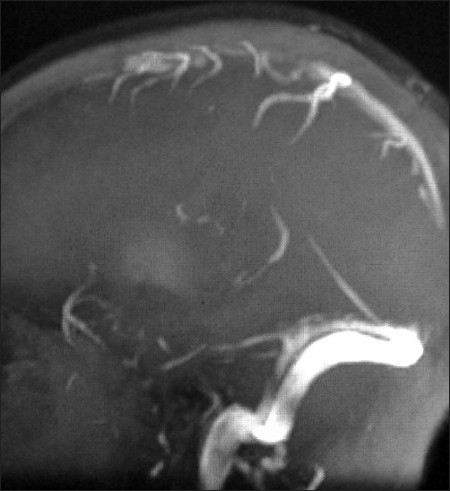
Maximum-intensity projection (MIP) MR venography image shows nonvizualization of the anterior portion of the superior sagittal sinus due to thrombosis

A 13-year-old girl being treated for acute mixed phenotypic leukemia developed headache and an episode of focal seizure. She was being given induction therapy for leukemia, with L-asparaginase 10000 units, vincristine 2 mg, and daunorubicin 40 mg. The coagulogram was significantly deranged at the time of presentation: the PT was 57% of normal, APTT was 62 sec, and INR was 1.38. Brain imaging was performed with plain and contrast-enhanced MRI to look for possible intracranial complications of leukemia as the cause of the neurological manifestations. Initial noncontrast T2-weighted MR image revealed a small hyperintense lesion in the left high parietal region of the brain, predominantly involving the white matter [Figure 3]. Coronal plane contrast-enhanced T1-weighted image demonstrated a nonenhancing superior sagittal sinus with the empty delta sign [Figure 4]. On MR venography there was nonvisualization of the anterior portion of the superior sagittal sinus due to absent flow, confirming the presence of thrombosis [Figure 5]. The clinical history, coagulogram readings, and imaging findings were diagnostic of asparaginase-induced coagulopathy manifesting as dural sinus thrombosis. Anticoagulation was started with low molecular weight heparin and continued with oral warfarin. Coagulogram parameters normalized over 5 days of treatment and the patient made a gradual but full clinical recovery. Repeat MRI showed normal signal intensity and enhancement of the previously thrombosed superior sagittal sinus.

## Discussion

Cerebral events in leukemia may be due to ischemia, hemorrhage, infection, or spread of the primary disease to brain.[4] Venous thrombosis may cause as many as 30% of the acute central nervous system events in acute leukemias. The estimated risk of thrombosis during the treatment of ALL in children is about 5%. Thrombotic events mainly occur in the central nervous system and upper limbs. Most of the thrombotic events occur during the induction phase of leukemia treatment. This is because of more intense treatment during this initial phase and, more importantly, the disease being active at this stage, there is a large lymphoblast population undergoing cytolysis. Patients receiving postinduction treatment have more stable disease and less thrombotic risk.[5] Drugs that enhance the risk of thrombosis include, most importantly, L-asparaginase and steroids.

L-asparaginase is established as an important treatment component during remission induction therapy of children with ALL.[6] L-asparaginase hydrolyses asparagine to aspartic acid and ammonia, and this may affect important proteins in the body. The changes affecting the proteins of the blood coagulation system have considerable clinical impact as they may induce bleeding as well as thromboembolic events.[5–7] Life-threatening complications may result when the central nervous system is involved. L-asparaginase may impair the hemostatic system by reducing the synthesis of coagulation factors (including fibrinogen, factor II, IX, and X) and inhibitors of coagulation (such as antithrombin, protein C, and protein S) as a consequence of asparagine depletion.[5–7] Despite a reduction of both procoagulant and anticoagulant activity, the hemostatic balance appears to be shifted towards a hypercoagulable state.[7] Asparaginase-induced deficiency of antithrombin III, the most important endogenous anticoagulant, significantly increases the risk of sinovenous thrombosis in the brain.[6] About 2% of children treated with L-asparaginase develop hemorrhagic or nonhemorrhagic infarcts consequent to CSVT.

The clinical manifestations of CSVT are variable and include headache, vomiting, altered mental status, focal deficits, and seizures.[1,7] The underlying pathology responsible for these symptoms is the spectrum of unilateral and bilateral venous infarcts and hemorrhages. Infarction and tissue damage result from decreased blood flow as a result of elevated retrograde venous pressure.[4,8] The evaluation of children with suspected CSVT has been made considerably easier by the modern neuroimaging techniques of CT and MRI. If emergency imaging ofthe venous sinuses is not undertaken the diagnosis is verylikely to be missed in affected children.[8]

Thrombosed sinus may appear hyperdense on noncontrast CT. Contrast-enhanced CT reveals enhancement around the thrombosed sinus in the form of the empty delta sign. Parenchymal infarcts in the distribution of the thrombosed draining vein/sinus, with or without hemorrhage, are additional findings on CT.[4,8] However, CT scan with contrast missesthe diagnosis of CSVT in up to 40% of patients. Early CT in case 1 of our series missed dural sinus thrombosis; when it was subsequently identified on the repeat CT, the damage had already been done. MRI is more sensitive for detection of early infarction.[9] Case 2 exemplifies this, with postcontrast MRI and MR venography identifying dural sinus thrombus when only subtle infarction was visible on plain CT. CT venography or MRI with venous MR(MRV) are now the methods of choice for investigation of CSVT.[10] The diagnosis is established by demonstratinga lack of flow in the dural sinuses and cerebral veins, with or without typicalimages of brain infarcts. Parenchymal MR and MRV are importantin the demonstration of both the infarct and the thrombus withinthe sinuses.[9,10] On MRI, the thrombus is readily recognizable inthe subacute phase, when it is of high signal intensity on T1-weightedimages; MRV is then often not required. In the acute phase, thethrombus shows isointense signal intensity on T1-weighted and low signalon T2-weighted imaging. This can be mistaken for flowing bloodbut MRV will demonstrate an absence of flow in the thrombosedsinus.[9,10]

Treatment of CSVT resulting from L-asparaginase-induced antithrombin deficiency includes general supportive measures, anticonvulsants for seizures, and anticoagulation.[1,5,7] L-asparaginase may be stopped for some time.[6] However, the key to management is early diagnosis by imaging as delayed institution of anticoagulation may be futile as in case 1. Rapid replenishment of coagulation factors may be achieved with fresh frozen plasma; antithrombin concentrates are preferred for this.[6] For therapeutic anticoagulation, low molecular weight heparin is given initially and this may be continued or it may be substituted by oral anticoagulants for 3–6 months.[1,5,7,8]

We conclude that, diagnosis of CSVT in leukemic patients being treated with L-asparaginase requires a high index of clinical suspicion in the presence of seizures, a focal neurological deficit, and features of raised intracranial tension. Early diagnosis demands a low threshold for imaging, and MRI should be preferred over CT. Identification of relevant findings such as venous infarcts, the empty delta sign, and absent flow in the dural sinuses on CT and MR venography enables proper diagnosis and management.
